# Femoroazetabuläres Impingement-Syndrom bei Adoleszenten – Wie beraten? Wie behandeln?

**DOI:** 10.1007/s00132-022-04214-z

**Published:** 2022-02-15

**Authors:** Catharina Chiari, Marie-Christine Lutschounig, Iris Nöbauer-Huhmann, Reinhard Windhager

**Affiliations:** 1 Universitätsklinik für Orthopädie und Unfallchirurgie, Klinische Abteilung für Orthopädie, Medizinische Universität Wien, Währinger Gürtel 18–20, 1090 Wien, Österreich; 2 Universitätsklinik für Radiologie und Nuklearmedizin, Abteilung für Neuroradiologie/Muskuloskelettale Radiologie, Medizinische Universität Wien, Wien, Österreich

**Keywords:** Arthroskopie, Hüfte, Schmerzen, Return to sport, Teenager, Arthroscopy, Hip, Pain, Return to sport, Teenagers

## Abstract

**Hintergrund:**

Das Femoroazetabuläre Impingement-Syndrom (FAIS) ist eine relevante Ursache für Leistenschmerzen beim Jugendlichen. Insbesondere sind Sportler betroffen.

**Ziel der Arbeit:**

Die Arbeit soll einen evidenzbasierten Hintergrund für Beratung und Therapie des FAIS beim Adoleszenten bieten.

**Material und Methoden:**

Anhand der aktuellen Literatur wurde eine Übersicht zu Prävalenz und Pathogenese, Abklärung und Diagnostik sowie therapeutischen Empfehlung des FAIS beim Adoleszenten erarbeitet.

**Ergebnisse und Diskussion:**

Das FAIS beim Jugendlichen betrifft vor allem sportlich aktive Patienten. Bestimmte Sportarten begünstigen die Entstehung eines FAIS. Cam-Impingement, Pincer-Impingement und kombiniertes FAIS sind die häufigsten Entitäten in dieser Altersgruppe. Die Cam-Morphologie entsteht kurz vor Schluss der proximalen Femurwachstumsfuge. Beim Cam-Impingement muss die Epiphyseolysis capitis femoris (ECF) von der primären Cam-Morphologie unterschieden werden. Die ECF verlangt eine rasche operative Versorgung mit Stabilisierung der Epiphyse, während das primäre Cam-Impingement elektiv abgeklärt werden kann und ein konservativer Behandlungsversuch sinnvoll ist. Schäden an Labrum und Knorpel werden regelhaft beobachtet. Eine systematische radiologische Abklärung mittels Projektionsröntgen und MRT ist obligat, um einen adäquaten Therapieplan zu entwickeln. Bei jugendlichen Patienten mit FAIS sollte immer ein konservativer Therapieversuch erfolgen. Ist dieser nicht erfolgreich, ist die operative Sanierung mit Hüftarthroskopie indiziert. Die postoperativen Ergebnisse zeigen bei Jugendlichen sehr gute Erfolge mit rascher Besserung der Beschwerden, geringen Komplikationen und einer hohen „Return-to-sport“-Rate.

## Abstract (en)

**Background:**

Femoroacetabular impingement syndrome (FAIS) is a relevant cause of groin pain in adolescents. Athletes are particularly affected.

**Objectives:**

The article shall provide an evidence-based background for FAIS counseling and therapy in adolescents.

**Material and methods:**

On the basis of the current literature, an overview of the prevalence and pathogenesis, evaluation and diagnostics, as well as the therapeutic recommendations for FAIS in adolescents was compiled.

**Results and discussion:**

FAIS in adolescents primarily affects physically active patients. Certain sports favor the development of FAIS. Cam impingement, pincer impingement, and combined FAIS are the most common entities in this age group. Cam morphology occurs shortly before closure of the proximal femoral growth plate. In cam impingement, the slipped capital femoral epiphysis (SCFE) must be distinguished from the primary cam morphology. SCFE requires rapid surgical treatment with stabilization of the epiphysis, while primary cam impingement can be analyzed electively, and conservative treatment is first recommended. Damage to the labrum and cartilage is regularly observed. A systematic radiological evaluation using X‑rays and MRI is mandatory in order to develop an adequate treatment plan. In adolescent patients with FAIS, a conservative attempt at therapy should always be made; if this is unsuccessful, surgical repair with hip arthroscopy is indicated. The postoperative results are very good in adolescents, with a rapid improvement in symptoms, few complications, and a high return-to-sport rate.

Die Behandlung des FAIS bei Adoleszenten stellt uns vor besondere Herausforderungen. Solange die Wachstumsfuge am Femurkopf noch nicht geschlossen ist, kann der Hüftkopf Formveränderungen erfahren. Dies gilt für die Entstehung einer Hüftkopfdeformität und für eine potenzielle Remodellierung von geringen Fehlstellungen. In dieser vulnerablen Phase sind Therapieentscheidungen besonders schwierig. Beim Jugendlichen hat die konservative Therapie einen größeren Stellenwert als beim Erwachsenen. Trotzdem muss die Indikationsstellung für die operative Behandlung konsequent genug gestellt werden.

## Prävalenz und Pathogenese

Das FAIS kann durch Cam, Pincer oder gemischte Formen ausgelöst werden. Die Angaben zur Prävalenz sind variabel. Kaymakoglu et al. analysierten CT-Bilder von 265 asymptomatischen Jugendlichen im Alter von 9–19 Jahren und fanden 26,5 % Cam, 17,6 % Pincer und 4,9 % gemischte Morphologien. In einer ähnlichen Untersuchung von Li et al. fanden sich 16,8 % Cam, 32,4 % Pincer und 6,1 % gemischte Morphologien. Die Cam-Morphologien überwiegen beim männlichen Geschlecht und treten bereits früher auf, wohingegen die Pincer-Morphologien mit zunehmendem Alter häufiger werden, öfter bei Frauen vorkommen und langsamer progredient sind. Die Beschwerden treten meist erst im Erwachsenenalter auf. Die Verteilung der FAIS-Typen ist vergleichbar zu jenen der adulten Population [10, 12].

**Figure 1:**
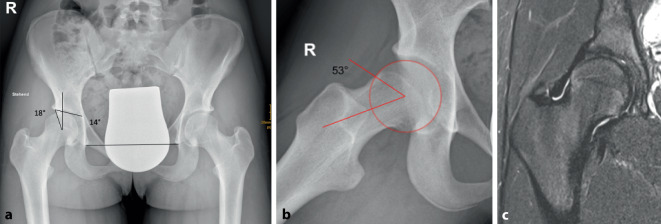

Die Genese der Cam-Morphologie ist im Jugendalter klinisch relevanter und deutlich besser untersucht als jene der Pincer-Morphologie. Wesentlich ist, dass die primäre von der sekundären Cam-Morphologie unterschieden werden muss. Dijkstra et al. führten erstmals eine Konzeptanalyse zur primären Cam-Morphologie durch, mit dem Ziel, Klarheit in Terminologie und Definition in das Konzept der Cam-Morphologie zu bringen. Dabei wurde definiert, dass sich die primäre Cam-Morphologie wahrscheinlich während der skelettalen Reifung bei jungen Adoleszenten (ohne vorhandene oder vorangegangene Hüfterkrankung) als physiologische Antwort auf stark belastende sportliche Aktivität und andere noch nicht bestätigte Risikofaktoren entwickelt. Diese beeinflussen die Wachstumsfuge des Hüftkopfes und führen zu einer epiphysären Hypertrophie und/oder epiphysären Extension [7]. Bei Athleten besteht eine Dosis-Wirkungs-Beziehung zwischen sportlicher Aktivität und Entstehung des Cams. Männer sind häufiger betroffen, Kapron et al. konnten jedoch auch für Athletinnen im College-Alter (Fußball, Volleyball, Leichtathletik) eine erhöhte Prävalenz nachweisen [9]. Zu den Sportarten, die mit einer besonders hohen Wahrscheinlichkeit von Cam-Morphologien assoziiert sind, zählen Fußball, Basketball, Volleyball, Eishockey, Tanzen, Gymnastik und Leichtathletik [5, 21]. Ein Fallbeispiel einer Tänzerin ist in Abb. 1 dargestellt.

**Figure 2:**
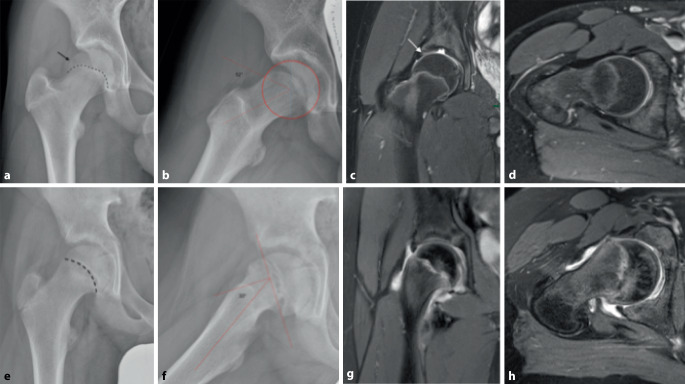

Die Cam-Morphologie kann bereits mit 10 Jahren auftreten und ist bei skelettaler Unreife zum Teil knorpelig angelegt, weshalb MRT-Untersuchungen aussagekräftiger als Röntgenbilder sind [25]. Zahlreiche Studien belegen, dass die Entstehung in den Jahren vor Schluss der Wachstumsfuge stattfindet und nach Fugenschluss nicht weiter fortschreitet [1, 19, 27]. Die häufigste Lokalisation ist anterosuperior am Schenkelhals, oft sind beide Hüften betroffen. Wie bereits weiter oben erwähnt, kommt es radiologisch zum Bild der epiphysären Extension (Abb. 2a,c), die prädiktiv für die Entwicklung einer Cam-Morphologie ist [20, 28]. Eine mögliche Erklärung ist, dass es sich um eine natürliche Reaktion auf Scherkräfte und juxtaphysäre Mikrotraumata handelt, die zu einem sogenannten Cupping-Phänomen führen, das wiederum einen Stabilisierungsversuch darstellt. Dies läuft parallel zum Verlust des epiphysären Tuberkels, der eine Rolle für die Stabilität der Epiphyse spielt, ab [15]. Die Quantifizierung der Cam-Morphologie erfolgt durch Messung des Alpha-Winkels nach Nötzli auf Röntgenbildern (Abb. 2b,d), CT oder MRT [22]. Die Cam-Morphologie muss nicht zwingend symptomatisch werden. Es ist weiterhin nicht geklärt, welche Patienten mit Cam-Morphologie Hüftschmerzen entwickeln. Eine herabgesetzte Innenrotation wurde als Risikofaktor beschrieben [11]. Ein niedriger Tönnis-Winkel, sowie eine azetabuläre Retroversion dürften ebenfalls ungünstige Faktoren darstellen [16]. Prospektive Studien zur endgültigen Definition von Risikofaktoren für die Entwicklung der Koxarthrose fehlen [26].

Wesentlich ist es, sekundäre Cam-Deformitäten von der primären Cam-Morphologie abzugrenzen

Wesentlich ist es, sekundäre Cam-Deformitäten von der primären Cam-Morphologie abzugrenzen, da sie sich hinsichtlich der Therapie unterscheiden. Vielfach wird die Epiphyseolysis capitis femoris (ECF) (engl. „slipped capital femoral epiphysis“, SCFE) als Ursache für eine Cam-Morphologie gesehen und mit der primären Cam-Morphologie gleichgesetzt. Post-ECF-Deformitäten weisen jedoch Unterschiede zur primären Cam-Morphologie auf. Die epiphysäre Extension fehlt, wohingegen eine vermehrte Verkippung (engl. „tilt“) der Epiphyse nach posterior vorhanden ist (Abb. 2e–h). Dies tritt gehäuft bei Jugendlichen mit erhöhtem BMI auf, wie in einer Studie asymptomatischer Patienten nachgewiesen wurde [23]. Wird eine solche Deformität in einer symptomatischen Hüfte eines jugendlichen Patienten detektiert, stellt sie eine dringende Indikation für eine chirurgische Behandlung dar, da die Epiphyse durch Stabilisierung vor einem zunehmenden Abrutsch geschützt werden muss. Auch nach einer Operation, insbesondere nach „in situ pinning“, behalten diese Hüften eine sekundäre Cam-Deformität, die zum Teil durch „remodelling“ gebessert wird, aber jedenfalls einer genauen Beobachtung bedarf [17]. Andere sekundäre Ursachen eines FAIS stellen Folgezustände nach Morbus Perthes, Frakturen oder entzündliche Gelenkerkrankungen dar. Auch extraartikuläre Impingement-Formen, wie das subspinale Impingement nach Avulsionsfrakturen oder Apophysenverletzungen, können beim jugendlichen Patienten vorkommen. Weitere Differenzialdiagnosen für den Hüftschmerz in dieser Altersgruppe sind Stressfrakturen, Hüftdysplasie oder Psoasschnappen (Coxa saltans interna). Nicht zuletzt müssen auch Torsionsfehler des Femurs berücksichtigt werden.

## Diagnostik

Die klinische Untersuchung wird analog zum erwachsenen Patienten durchgeführt. An dieser Stelle kann nur eine Zusammenfassung der wichtigsten Untersuchungstechniken gegeben werden. Dazu gehören die Beurteilung des Gangbildes (Hinken, Einwärtsgang, Auswärtsgang), die Untersuchung des Hüftgelenks mit Erhebung des Bewegungsumfanges in Rücken- und Bauchlage und die Durchführung der klassischen Impingement-Tests in Flexion‑, Adduktion- und Innenrotation (FADIR), sowie in Flexion-Abduktion und Außenrotation (FABER). Beim jugendlichen Patienten sollte insbesondere das Drehmann-Zeichen (spontane Außenrotation bei zunehmender Beugung im Hüftgelenk) berücksichtigt werden, da es einen Hinweis auf das Vorliegen einer ECF gibt. Durch Palpation werden Schmerzdruckpunkte in der Leiste und am Trochanter festgestellt. Spezielle Funktionstest können beispielsweise bei Schnappen der Psoassehne (Heben und Absenken des gestreckten Beines, kreisende Bewegungen des gestreckten Beines) hilfreich sein [29].

Die weitere Abklärung erfolgt radiologisch. Standard ist die Beckenübersichtsaufnahme im Liegen und die Aufnahme in einer zweiten Ebene. Dazu wird eine axiale Aufnahme durchgeführt. Die Cam-Morphologie wird am besten in der Dunn-Aufnahme dargestellt, bei Verdacht auf ECF sollte das Ausmaß des Abrutsches in der Lauenstein-Aufnahme vermessen werden, die Hüftgelenksdysplasie wird mit einer Faux-profil-Aufnahme zur Beurteilung der vorderen Überdachung abgeklärt. Die Faux-profil-Aufnahme eignet sich jedoch auch zur Darstellung eines subspinalen Impingements oder der prominenten vorderen Überdachung beim Pincer-Impingement. Eine Ganzbeinaufnahme im Stehen kann ergänzend sinnvoll sein, wenn Beinachsendeformitäten oder Beinlängendifferenzen analysiert werden sollen. Die MRT zählt mittlerweile ebenfalls zum Goldstandard. Durch radiäre Rekonstruktion können Ausmaß und Lokalisation einer Cam-Morphologie nach der Ziffernblattmethode beurteilt werden. Indirekte Zeichen eines Impingements können Knochenmarksödeme im Bereich der Kontaktzonen sein. Wesentlich ist auch die Beurteilung des Labrums und des Knorpels. Im Erwachsenalter wird idealerweise eine MR-Arthrographie (optional mit Traktion) durchgeführt, die eine detaillierte Darstellung der Knorpeloberfläche und des Labrums erlaubt. Aufgrund der Invasivität der Untersuchung ist diese Technik jedoch für Kinder und Jugendliche nicht routinemäßig anwendbar.

Die Beurteilung von Sekundärschäden an Knorpel und Labrum ist für die Operationsindikation und -planung von Bedeutung [2]. In einer Studie von Lieberman et al. wurde der intraoperative Befund von Patienten mit Post-ECF-Impingement und primärer Cam-Morphologie verglichen. In beiden Gruppen wurden Knorpelschäden festgestellt. Es konnte gezeigt werden, dass die Deformitäten nach ECF einen deutlich höheren Alpha-Winkel aufwiesen, zu ausgedehnteren Knorpelschäden führten und die Patienten deutlich jünger waren [13]. Diese Resultate werden auch durch eine Studie von Örtegren et al. unterstrichen, die den Knorpelstatus mit dGEMRIC(„delayed gadolinium-enhanced magnetic resonance imaging of cartilage“)-MRT bei jungen Patienten nach ECF-„pinning“ untersuchten. Hier konnte eine Knorpeldegeneration in Abhängigkeit vom Alpha-Winkel nachgewiesen werden, unabhängig vom initialen Abrutschwinkel. Die Autoren empfehlen demnach, Patienten nach ECF möglichst bald nach Fugenschluss einer MRT-Diagnostik zu unterziehen [24]. Youngman et al. untersuchten intraoperativ das Ausmaß von Knorpel- und Labrumschäden bei adoleszenten FAIS-Patienten und fanden Labrumschäden bei 88,4 %. Ein höherer Alpha-Winkel korrelierte mit dem Ausmaß des Labrumschadens [30]. Somit sollte auch bei Adoleszenten mit FAIS besonders Wert auf eine MRT-basierte Knorpeldiagnostik gelegt werden, um früh genug zu intervenieren. Am Zentrum der Autoren dieser Arbeit wird routinemäßig bei jeder Hüft-MRT mit der Verdachtsdiagnose eines Impingements oder einer Hüftdysplasie auch eine Torsionsanalyse mit Rotations-MRT durchgeführt. Torsionsfehler sind ebenfalls in die Therapieplanung miteinzubeziehen.

## Therapie – wie beraten, wie behandeln?

Im Vergleich zum Erwachsenenalter hat die konservative Therapie bei der Behandlung des primären FAIS des Adoleszenten einen höheren Stellenwert. In einer rezenten prospektiven Studie berichten Zogby et al. über 5‑Jahres-Resultate der nichtoperativen Therapie von FAIS bei Adoleszenten. Das Einschlusskriterium in die Studie waren Leistenschmerzen sowie ein positiver anteriorer Impingement-Test. Es erfolgte eine radiologische Abklärung mit Nativröntgen in zwei Ebenen. Nur ein Teil der Patienten wurde mit MRT abgeklärt. 69 Hüftgelenke von 51 Patienten wurden nach 5 Jahren untersucht. Nur 3 Patienten übten keinen Sport aus, alle anderen waren sportlich aktiv. 46 % hatten ein Cam-Impingement, 13 % ein Pincer-Impingement und 14 % ein kombiniertes Impingement. 26 % hatten keine radiologischen Zeichen eines Impingements. Die konservative Behandlung bestand in einer Sportpause von 6 Wochen für Sportarten, die Laufen, Springen oder hochgradige Hüftbeugung inkludierten. Begleitend wurde Physiotherapie durchgeführt, wobei hier keine detaillierten Angaben zur Art der Therapie gemacht wurden. Jene Patienten, die keine Besserung erfuhren, erhielten eine intraartikuläre Injektion mit 40 mg Glukokortikoid (Triamcinolon) mit Lokalanästhetikum, jene, die die Injektion ablehnten oder weiter Beschwerden hatten, wurden arthroskopiert. Jene Patienten, die aufgrund von therapieresistenten Beschwerden einer MRT zugeführt wurden hatten zu 70 % einen Labrumriss. Nach 1, 2 und 5 Jahren wurden „patient reported outcomes“ (PRO), nämlich der modified Harris Hip Score (mHHS) und der Nonarthritic Hip Score (NAHS) erhoben. 72 % wurden erfolgreich mit Aktivitätsreduktion und Physiotherapie behandelt, 10 % erhielte eine Injektion ohne nachfolgende Operation und 17 % wurden arthroskopiert. Die Verbesserung der PRO war innerhalb der ersten beiden Jahre signifikant, danach bleiben die Ergebnisse stabil. Es gab keine Unterschiede zwischen den Behandlungsgruppen. Alle bis auf eine Operation wurde innerhalb der ersten beiden Jahre nach Behandlungsbeginn durchgeführt. 71 % kehrten zu ihrer sportlichen Aktivität zurück. Trotz methodischer Limitationen zeigte diese Arbeit, dass die nichtoperative Therapie in der adoleszenten FAIS-Population einen wichtigen Stellenwert hat und zu einer stabilen Verbesserung der klinischen Symptomatik über 5 Jahre bei der Mehrheit der untersuchten Patienten führte [31].

Eine andere Studie, die sich mit konservativer Therapie von Labrumrissen beschäftigt, wurde von Cianci et al. publiziert. Es handelt sich um eine retrospektive Studie, die adoleszente Patienten untersuchte, die wegen Labrumrissen behandelt wurden. Von den 76 Patienten waren 62 weiblich. Die am häufigsten ausgeübten Sportarten waren Tanzen, Fußball und Gymnastik. 68,4 % wurden mit Physiotherapie, 72,4 % mit einer intraartikulären Injektion mit Triamcinolon und Ropivacain und 56,6 % mit einer Kombination aus beidem behandelt. 76,3 % der Patienten wurden in weiterer Folge operiert. 51,3 % der Patienten mit simultanem FAIS wurden operiert, während nur 25 % der Patienten mit Labrumriss ohne FAIS operiert wurden. Der Anteil der Hüftdysplasiepatienten wurde allerdings nicht beschrieben. In dieser Patientenkohorte war die konservative Therapie nur über einen kurzen Zeitraum erfolgreich [5]. Es muss berücksichtigt werden, dass die Patienten in dieser Studie eine sehr spezielle Population darstellen und die Ergebnisse kritisch interpretiert werden müssen.

Zur operativen Therapie des FAIS beim Jugendlichen existieren deutlich mehr Daten als zur konservativen Therapie

Deutlich mehr Daten existieren zur operativen Therapie des FAIS beim Jugendlichen. In den USA nahm die Anzahl an Hüftarthroskopien bei jugendlichen Patienten in einem Zeitraum von 10 Jahren (2008 bis 2018) um das 3,9-Fache zu [8]. Eine Metaanalyse zum arthroskopischen Management von FAIS bei Jugendlichen analysierte die Daten von 406 Adoleszenten (53 % weiblich) mit einem durchschnittlichen Alter von 15,9 Jahren und Follow-up von 30,4 Monaten. Die Ergebnisse waren sehr positiv: 94 % hatten ihre sportliche Aktivität wieder erreicht, die subjektiven Scores zeigten eine signifikante Verbesserung. Die Komplikationsrate lag bei 1,1 % (temporäre Parästhesien im Versorgungsgebiet des N. cutaneus femoris lateralis oder perineal), die Revisionsrate bei 5 % (Adhäsionen zwischen Kapsel und Labrum, Rezidiv der Cam-Deformität, Verletzungen, unklare Ursachen) [18].

Eine weitere Metaanalyse von 618 Adoleszenten mit Durchschnittsalter 15,8 Jahren und 57 % Frauenanteil zeigte ebenfalls, dass die arthroskopische Operation beim FAIS zu einer hohen Patientenzufriedenheit und verbesserter Funktion und Rückkehr zum Sport führte. Die häufigste Diagnose (64 %) war ein kombiniertes Cam- und Pincer-Impingement, die Komplikations- und Reoperationsrate war sehr niedrig [4].

Litrenta et al. konnten auch zeigen, dass nach 2 Jahren eine hohe „Return-to-sport“-Rate mit geringen Komplikationen erzielt werden konnte. In dieser Patientenserie von 96 Patienten (75,3 % weiblich) mit einem Durchschnittsalter von 15,9 Jahren waren Labrumrekonstruktionen (81,5 %) am häufigsten, danach folgten Ileopsoassehnenverlängerungen (72,8 %), Offsetkorrekturen am Schenkelhals (69,1 %) und Azetabuloplastiken (66,7 %). Die Kapsel wurde bei 86,4 % verschlossen oder gerafft. In diesem Zusammenhang wurde erwähnt, dass eine große Anzahl der weiblichen Patienten einen erhöhten Beighton-Score zeigten und somit der Kapselverschluss zur Prävention der Hüftinstabilität essenziell ist. In dieser Studie wurden auch die Knorpel- und Labrumschäden intraoperativ detailliert dokumentiert. 23,5 % hatten Knorpelschäden Grad 2 oder mehr, 4,9.% hatten Knorpelschäden am Hüftkopf [14]. Bemerkenswert ist die hohe Anzahl an Ileopsoassehnenverlängerungen in dieser Kohorte, die bei Patienten mit schmerzhaftem Schnappen durchgeführt wurden, die möglicherweise auf die Patientenselektion zurückzuführen ist, da die Eingriffe an einem hochspezialisierten Zentrum durchgeführt wurden.

Ein Thema, das bei Patienten mit offener Wachstumsfuge des Hüftkopfes diskutiert wird, ist das potenzielle Risiko eines Rezidivs der Cam-Morphologie nach Cam-Abtragung. In einer Studie von Arashi et al. wurden Patienten mit skelettaler Unreife (offen Physe des proximalen Femurs oder Risser-Stadium ≤ 4) mit Erwachsenen vergleichen. Bei 4 von 27 Hüften kam es zu einem Wiederauftreten der Cam-Morphologie, die eine neuerliche Arthroskopie erforderte. Alle Patienten waren männlich und circa 15 Jahre alt. Die Autoren stellen fest, dass das Cam-Rezidiv in dieser Patientengruppe ein Risiko darstellt, das nicht negiert werden sollte [3].

### Infobox Sportarten mit hohem Risiko für FAIS bei Adoleszenten [4, 6, 9]

- Fußball
- Eishockey
- Leichtathletik
- Tanz, Gymnastik
- Basketball
- Volleyball

## Fazit für die Praxis

- Bei jugendlichen Patienten mit Leistenschmerzen ist das FAIS (Femoroazetabuläres Impingement-Syndrom) immer eine häufige Differenzialdiagnose. Sportlich sehr aktive Patienten sind am häufigsten betroffen.
- Die Cam-Morphologie ist beim jugendlichen Patienten vorherrschend und entsteht kurz vor Verschluss der Wachstumsfuge am proximalen Femur.
- Die primäre Cam-Morphologie ist von der sekundären Cam-Morphologie bei ECF (Epiphyseolysis capitis femoris) abzugrenzen. Die ECF darf als Differenzialdiagnose nicht übersehen werden, da sie eine akute Operationsindikation darstellt. Post-ECF-Deformitäten sind mit einem größeren Alpha-Winkel assoziiert, treten bei jüngeren Kindern mit erhöhtem Body-Mass-Index auf und führen rascher zu Labrum- und Knorpelschäden.
- Eine systematische radiologische Abklärung mit Röntgen in 2 Ebenen (Beckenübersicht, Dunn-Aufnahme) und MRT ist obligat.
- Das symptomatische FAIS beim Jugendlichen sollte primär einem konservativen Therapieversuch zugeführt werden. Dieser beinhaltet Sportkarenz von 6 Wochen bis 3 Monate, Vermeidung von maximalen Beuge- und Rotationsbewegungen und gegebenenfalls einer intraartikulären Injektion mit Cortison und Lokalanästhetikum (Ropivacain).
- Die Hüftarthroskopie ist bei adoleszenten Sportlern mit FAIS ein sehr erfolgreicher Eingriff, mit signifikanter Verbesserung der klinischen Symptome und hohen „Return-to-sport“-Raten.

## Abkürzungen

BMI: Body-Mass-Index
dGEMRIC: „Delayed gadolinium-enhanced magnetic resonance imaging of cartilage“
ECF: Epiphyseolysis capitis femoris
FABER: Flexion-Abduktion und Außenrotation
FADIR: Flexion‑, Adduktion- und Innenrotation
FAIS: Femoroazetabuläres Impingement-Syndrom
mHHS: Modified Harris Hip Score
NAHS: Nonarthritic Hip Score
PRO: „Patient reported outcomes“
SCFE: „Slipped capital femoral epiphysis“
